# Multicenter comparative analysis of preoperative and postoperative outcomes after holmium laser enucleation of the prostate based on the prostate volume

**DOI:** 10.20452/wiitm.2025.17955

**Published:** 2025-05-29

**Authors:** Samer Al‑Rawashdah, Malik Ayyad, Khalil Abu Zahra, Saddam Al Demour, Mohammad Al‑Zubi, Omar Ayaad

**Affiliations:** Urology Unit, Department of Special Surgery, Faculty of Medicine, Mutah University, Karak, Jordan; Urology Department, Specialty Hospital, Amman, Jordan; Division of Urology, Department of Special Surgery, School of Medicine, University of Jordan, Amman, Jordan; Urology Division, Surgery and Anesthesia Department, Faculty of Medicine, Yarmouk University, Irbid, Jordan; Quality and Accreditation Department, Sultan Qaboos Comprehensive Cancer Care and Research Center, University Medical City, Muscat, Jordan

**Keywords:** benign prostatic hy‑ perplasia, holmium laser enucleation of the prostate, prostate volume, quality of life, surgical outcomes

## Abstract

**INTRODUCTION:**

Holmium laser enucleation of the prostate (HoLEP) is considered the best treatment for benign prostatic hyperplasia (BPH). This procedure offers effective symptom relief across varying prostate sizes.

**AIM:**

This study aimed to evaluate preoperative and postoperative outcomes of HoLEP in patients with different prostate volumes in a multicenter comparative analysis conducted in Jordan.

**MATERIALS AND METHODS:**

A cohort study was conducted in 3 private clinics in Amman, Jordan. It included 77 patients who were divided into 2 groups according to prostate volume: smaller than or equal to 40 ml (n = 37) and larger than 40 ml (n = 40). The data collected preoperatively and at 3 months postoperatively included urinary symptoms, quality of life (QoL) scores, and functional parameters. Statistical analyses included paired *t* tests and independent *t* tests.

**RESULTS:**

Both groups exhibited improvements in relation to initial values, as assessed after the procedure using the International Prostate Symptom Score (≤40 ml, 16 vs 7.6; >40 ml, 14.9 vs 4.6; *P *<⁠0.001). Their QoL scores also improved (≤40 ml, 3.2 vs 1.5; >40 ml, 3 vs 1.3; *P *<⁠0.001). The maximum urinary flow rate increased in both groups (≤40 ml, 8.2 ml/s vs 14 ml/s; >40 ml, 8.6 ml/s vs 13.6 ml/s; *P *<⁠0.001), with similar improvements in postvoid residual urine volume. Larger prostates required longer enucleation times (≤40 ml, 19.8 min; >40 ml, 39.3 min; *P *= 0.04) with more tissue removal (≤40 ml, 4.7 g; >40 ml, 17 g; *P *<⁠0.001).

**CONCLUSIONS:**

HoLEP effectively improved various measured parameters, including urinary symptoms, QoL, and functional outcomes in patients with BPH across all prostate sizes. Larger prostates required procedures of increased complexity.

## INTRODUCTION

Benign prostatic hyperplasia (BPH) is one of the most common urological conditions affecting aging men worldwide. The prevalence of BPH increases with age, affecting up to 50% of men aged 50–60 years and over 80% of men aged 70–80 years.^1^ BPH is characterized by lower urinary tract symptoms, such as increased urination frequency and urgency, nocturia, and incomplete bladder emptying.^1^**^,^**^2^

**FIGURE 1:**
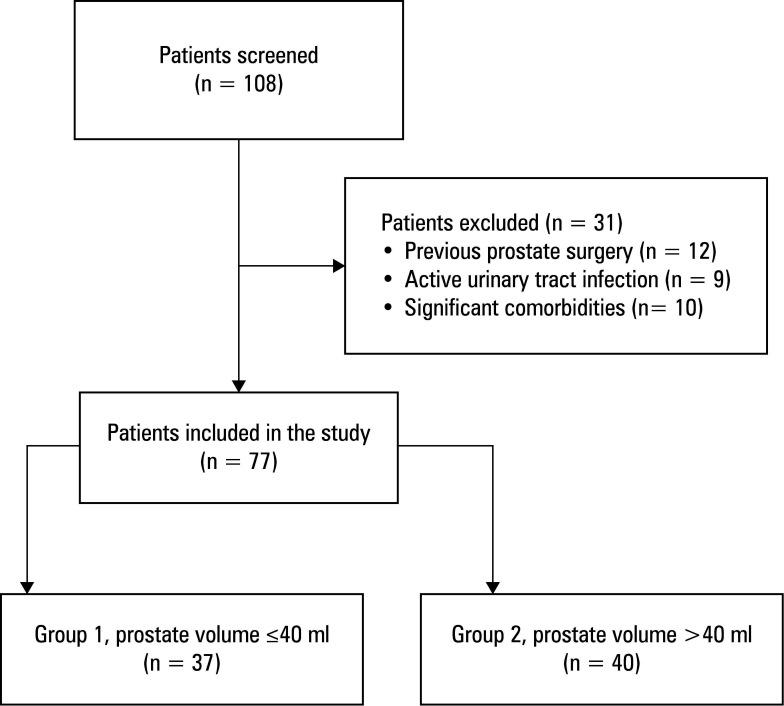
Flow chart of patient selection and group allocation for holmium laser enucleation of the prostate

BPH is a progressive condition. In many patients, symptom severity worsens over time.^2^ The progression of BPH may require multiple interventions. This causes an increasing cumulative burden, since managing comorbidities in aging patients complicates treatment decisions.^1^**^,^**^2^

Early diagnosis and intervention can improve patient outcomes and reduce long-term complications. The heterogeneity of BPH symptoms makes individualized treatment essential. Traditional surgical treatments, such as transurethral resection of the prostate (TURP), carry numerous risks, such as bleeding, infection, and prolonged recovery.^3^ Therefore, alternative approaches are needed to manage large prostate volumes.^4^

Nonsurgical interventions, such as the UroLift system (Teleflex Interventional Urology, Pleasanton, California, United States), are not suitable for all patients. Notably, treating patients with obstructions or larger prostates poses great challenges. In such cases, pharmacological treatments, such as α-blockers and 5-α reductase inhibitors, are used, but they often fail to provide complete symptom relief and may trigger various adverse effects, such as dizziness, hypotension, and sexual dysfunction. These issues can lead to poor adherence.^4^**^,^**^5^ Additionally, balancing symptom relief with the preservation of sexual function remains a challenge.^1^**^,^**^5^ This may lead to an increased demand for treatments that combine effectiveness with minimal invasiveness and quick recovery.

Holmium laser enucleation of the prostate (HoLEP) is a minimally invasive procedure, recognized for its adaptability across all prostate sizes. In fact, some patients benefit from it, regardless of volume considerations.^1^**^,^**^3^**^,^**^6^ While the laser helps remove blocking tissues via morcellation, some reports^1^**^,^**^7^ emphasize HoLEP’s reduced complication profile, as compared with open prostatectomy or TURP. It carries a lower risk of bleeding and reoperation.

Recovery times are frequently shorter, but it is not the only aspect patients seem to appreciate about HoLEP. Many actually report improved satisfaction, most likely due a longer absence of symptoms and a better overall quality of life (QoL).^8^**^,^**^9^ This method has also been increasingly used for large or more challenging prostates due to both its safety and versatility.^10^

Still, large prostate volumes introduce certain complexities. Studies suggest that larger sizes might translate into longer enucleation times, and possibly more tissue to remove.^11^**^,^**^12^ It can affect patients’ preoperative preparation and even influence the planning of the procedure.

Studies indicate that HoLEP brings substantial symptom relief for patients with a larger prostate.^13^**^,^**^14^ This improves patient expectation management, allowing for a better understanding of volume-related outcomes, which can guide future innovations and refinements in BPH treatment.^13^**^,^**^15^**^,^**^16^ However, the limited availability of comprehensive data makes it challenging to implement evidence-based national guidelines.

## AIM

The study aimed to evaluate preoperative and postoperative characteristics and outcomes of HoLEP patients with different prostate volumes.

## MATERIALS AND METHODS

### Setting and design

A comparative cohort study design was used to evaluate the characteristics and outcomes of HoLEP in patients with different prostate volumes. The study was conducted in 3 hospitals in Amman, Jordan, between January 2022 and December 2024.

### Patients

The study included 77 patients. Inclusion criteria comprised men diagnosed with BPH, aged 40 years or more, who underwent HoLEP. Exclusion criteria were previous prostate surgeries, active urinary tract infections, or significant comorbidities affecting surgical risk or outcomes. FIGURE 1  outlines the selection process. Out of 108 patients initially screened, 31 did not meet the inclusion criteria. The final sample consisted of 77 patients, who were then classified into 2 groups based on prostate volume: 37 patients with volume smaller than or equal to 40 ml (group 1) and 40 patients with volume greater than 40 ml (group 2).

### Intervention

All patients underwent HoLEP performed by experienced urologists using standardized surgical protocols. The procedure involved laser enucleation of obstructive prostate tissue, followed by morcellation for tissue removal. Preoperative and postoperative care was standardized for all patients.

### Outcome measures

The primary objective of this study was to evaluate the clinical effectiveness of HoLEP in improving urinary symptoms, functional voiding parameters, and patient-reported QoL. All primary outcomes were measured preoperatively and at 3 months postoperatively.

**TABLE 1:** Study variables and their definitions

Variable	Definition	Instrument/method	Reference
Prostate volume, ml	Prostate volume refers to the size of the prostate gland, measured in milliliters. It is typically calculated using TRUS based on the ellipsoid formula: volume = π/6 × height × width × length, where dimensions (mm) are obtained from axial and sagittal TRUS images.	TRUS	^17^
IPSS total score	A validated clinical tool used to assess the severity of lower urinary tract symptoms associated with BPH. It consists of 7 questions addressing both voiding symptoms (incomplete emptying, intermittency, weak stream, straining) and storage symptoms (frequency, urgency, nocturia). Each item is scored from 0 (not at all) to 5 (almost always), producing a total score ranging from 0 to 35, with higher scores indicating more severe symptoms. It is commonly used for diagnosis, monitoring treatment response, and comparing treatment outcomes in urological practice.	IPSS questionnaire	^18^
IPSS voiding subscore	It is a subtotal of 4 items: incomplete emptying, intermittency, weak stream, and straining.	IPSS items 1, 3, 5, 6	^18^
IPSS storage subscore	It is a subtotal of 3 items: frequency, urgency, and nocturia.	IPSS items 2, 4, 7	^18^
IPSS QoL score	It is a patient-rated symptom discomfort, scored from 0 (delighted) to 6 (terrible).	IPSS item 8	^18^
Qₘₐₓ, ml/s	It is the peak rate at which urine is expelled during voiding, measured using uroflowmetry. It is a key urodynamic parameter used to assess the degree of bladder outlet obstruction.	Uroflowmetry	^19^
PVR, ml	It is the volume of urine remaining in the bladder immediately after voluntary voiding.	Ultrasound bladder scan	^20^
Enucleation time, min	It refers to the time from the initial laser incision to the completion of prostate adenoma removal during the HoLEP procedure. It reflects the operative efficiency and complexity, often influenced by prostate size, surgeon experience, and the degree of prostatic hyperplasia.	Operating room timer; clinical surgical records	^1^,^14^
Enucleated tissue weight, g	It refers to the mass of the prostate adenoma tissue removed during the HoLEP procedure. It reflects the amount of prostatic tissue excised, often correlating with the preoperative prostate volume and severity of BPH.	Postoperative measurement	^14^,^21^
Enucleated tissue ratio, %	The enucleated tissue ratio is the percentage of the total prostate volume that was surgically removed during HoLEP, calculated as: (enucleated tissue weight/preoperative prostate volume) × 100.	Derived from operative and TRUS data	^14^,^15^, ^21^
Prostate-specific antigen, μg/l	It is a glycoprotein enzyme produced by epithelial cells of the prostate. It is commonly assessed via serum testing and used as a biomarker for evaluating prostate health, including the presence of BPH, prostatitis, or prostate cancer.	Laboratory blood test	^22^

Abbreviations: BPH, benign prostatic hyperplasia; HoLEP, holmium laser enucleation of the prostate; IPSS, International Prostate Symptom Score; PVR, postvoid residual urine volume; QoL, quality of life; Q_max_, maximum urinary flow rate; TRUS, transrectal ultrasonography

The outcome measurement instruments included: 1) International Prostate Symptom Score (IPSS): a validated tool measuring lower urinary tract symptoms, yielding 3 scores: total score; voiding subscore (incomplete emptying, intermittency, weak stream, straining); and storage subscore (urination frequency and urgency, nocturia)^2^**^,^**^6^**^,^**^18^; 2) QoL score derived from the IPSS questionnaire, evaluating the patient’s perceived discomfort from urinary symptoms on a scale from 0 (delighted) to 6 (terrible)^2^**^,^**^13^**^,^**^18^; 3) maximum urinary flow rate (Q_max_) measured in ml/s using uroflowmetry, reflecting the peak urinary stream rate^2^^,^^6^**^,^**^19^; and 4) postvoid residual urine volume (PVR) assessed via bladder ultrasonography, indicating the amount of urine left in the bladder after voiding.^2^**^,^**^6^**^,^**^20^

Secondary outcomes were measured to assess intraoperative and perioperative surgical efficiency and safety. These included changes in hemoglobin (Hb) levels, enucleation time, and the weight of enucleated tissue. Difference in Hb levels (g/l) before and after surgery served as an indicator of blood loss. Enucleation time, defined as the time from the initial laser incision to the completion of enucleation, was measured to reflect procedural complexity.**^1^** Total weight of the enucleated prostate tissue was recorded after morcellation, and the enucleated tissue ratio was calculated by dividing the enucleated tissue weight by the preoperative prostate volume and multiplying it by 100, to evaluate the completeness and efficiency of tissue removal.^14^**^,^**^21^ All data were consistently collected across the 3 participating centers using standardized protocols and instruments. TABLE 1 presents the study variables and their definitions.

### Statistical analysis

Patients were assigned into 2 groups based on prostate volume. Group 1 included participants with prostate volume smaller than or equal to 40 ml, and group 2 comprised patients with prostate volume greater than 40 ml. Data analysis was performed using IBM SPSS Statistics package version 21 (IBM Corp., Armonk, New York, United States). Prior to the analysis, all continuous variables were evaluated for normality using a visual assessment of histograms and quantile-quantile plots, and were found to follow an approximately normal distribution. The clinical characteristics of the 2 groups were evaluated using the *t *test for continuous variables (2-tailed tests). The data were reported as mean (SD). Within each group, changes in continuous variables were analyzed using paired (2-tailed) *t* tests to compare preoperative and postoperative results at follow-up after 3 months. Independent *t* tests were used to assess differences in changes between the groups. All statistical tests were 2-sided, with a *P* value below 0.05 deemed significant.

**TABLE 2:** Preoperative characteristics of the patients

Parameter	Group 1, prostate volume ≤40 ml (n = 37)	Group 2, prostate volume >40 ml (n = 40)	P value (2-tailed)
Age, y	54.9 (7.8)	54.9 (6.7)	0.99
IPSS QoLscore, points	3.2 (1.1)	3 (1.2)	0.44
IPSS total score, points	16 (7.2)	14.9 (6.6)	0.17
IPSS voiding subscore, points	10.5 (5.1)	8.8 (4.5)	0.19
IPSS storage subscore, points	5.7 (3.5)	6.1 (3.2)	0.04
PVR, ml	47.6 (58.7)	47.8 (58.2)	0.99
Qₘₐₓ, ml/s	8.2 (3.6)	8.6 (4.3)	0.68
Prostate volume on TRUS, ml	20 (2.8)	49.8 (19.7)	<0.001
Prostate-specific antigen, μg/l	1.3 (1.3)	2.5 (2)	0.007
BMI, kg/m²	21.2 (1.3)	20.3 (2)	0.06
Hb, g/l	123 (9)	117 (9)	0.03

Data are presented as mean (SD).

Abbreviations: BMI, body mass index; Hb, hemoglobin; others, see TABLE 1

**TABLE 3:** Improvements in key urinary parameters after holmium laser enucleation of the prostate in patients with prostate volume ≤40 ml

Parameter	Before HoLEP	After HoLEP	P value (2-tailed)
Hb, g/l	123 (9)	112 (12)	0.07
IPSS QoL score, points	3.2 (1.1)	1.5 (0.7)	<0.001
IPSS total score, points	16 (7.2)	7.6 (4.6)	<0.001
IPSS voiding subscore, points	10.5 (5.1)	3.2 (2.9)	<0.001
IPSS storage subscore, points	5.7 (3.5)	4.2 (2.2)	0.06
PVR, m/l	47.6 (58.7)	17 (18.4)	0.03
Qₘₐₓ, ml/s	8.2 (3.6)	14 (6.6)	<0.001

Abbreviations: see TABLE 1 and TABLE 2

**TABLE 4:** Improvements in key urinary parameters after holmium laser enucleation of the prostate in patients with prostate volume >40 ml

Parameter	Before HoLEP	After HoLEP	P value (2-tailed)
Hb, g/l	117 (9)	106 (12)	<0.001
IPSS QoL score, points	3 (1.2)	1.3 (1)	<0.001
IPSS total score, points	14.9 (6.6)	4.6 (3.3)	<0.001
IPSS voiding subscore, points	8.8 (4.5)	1.3 (1.7)	<0.001
IPSS storage subscore, points	6.1 (3.2)	3.3 (2.4)	<0.001
PVR, ml	47.8 (58.2)	21.3 (28.4)	<0.001
Qₘₐₓ, ml/s	8.6 (4.3)	13.6 (7.3)	<0.001

Abbreviations: see TABLE 1 and TABLE 2

### Ethics

The study adhered to the ethical principles outlined in the Declaration of Helsinki. Ethical approval was obtained from the institutional review board of Mutah University (21025). Data were anonymized to protect patient privacy.

## RESULTS

In TABLE 2, we compare preoperative characteristics between the 2 groups based on prostate volume (≤40 ml [n = 37] vs >40 ml [n = 40]). Both groups were similar in the majority of preoperative characteristics. Other variables, including age (54.9 vs 54.9 years), total IPSS score (16 vs 14.9), flow rates (8.2 vs 8.6 ml/s), prostate-specific antigen levels (1.3 vs 2.5 µg/l), body mass index (21.2 vs 20.3 kg/m²), and Hb levels (123 vs 117 g/l) were similar between the groups.

TABLE 3 illustrates improvements in key urinary parameters after HoLEP in group 1. Hb levels decreased slightly from mean (SD) 123 (9) to 121 (12) g/l (*P *= 0.07), indicating minimal impact of the procedure on Hb. The IPSS QoL score improved markedly from 3.2 (1.1) to 1.5 (0.7; *P *<⁠0.001), and toal IPSS score decreased considerably from 16 (7.2) to 7.6 (4.6; *P *<⁠0.001).

The voiding subscore improved substantially, dropping from 10.5 (5.1) to 3.2 (2.9; *P *<⁠0.001), while the storage subscore showed a modest decrease from 5.7 (3.5) to 4.2 (2.2; *P *= 0.06). Additionally, postvoid PVR decreased markedly from 47.6 (58.7) to 17 (18.4) ml (*P *= 0.03). Q_max_ increased considerably from 8.2 (3.6) to 14 (6.6) ml/s (*P *<⁠0.001).

TABLE 4 highlights improvements in urinary parameters after HoLEP in group 2. Hb levels showed a notable reduction from mean (SD) 117 (9) to 106 (12) g/l (*P *<⁠0.001), reflecting a significant impact of the procedure on hemoglobin. The IPSS QoL score improved markedly from 3 (1.2) to 1.3 (1; *P *<⁠0.001), while total IPSS score decreased substantially from 14.9 (6.6) to 4.6 (3.3; *P *<⁠0.001).

The voiding subscore showed a remarkable reduction from 88.8 (4.5) to 1.3 (1.7; *P *<⁠0.001), and the storage subscore also decreased notably from 6.1 (3.2) to 3.3 (2.4; *P *<⁠0.001). Furthermore, PVR decreased from 47.8 (58.2) to 21.3 (28.4) ml (*P *<⁠0.001), indicating improved bladder emptying. Q_max_ increased considerably from 8.6 (4.3) to 13.6 (7.3) ml/s (*P *<⁠0.001), reflecting enhanced urinary flow.

**TABLE 5:** Comparative analysis of outcomes of holmium laser enucleation of the prostate

Parameter	Group 1, prostate volume ≤40 ml (n = 37)	Group 2, prostate volume >40 ml (n = 40)	P value (2-tailed)
Hb, g/l, mean (SD)	7 (9)	11 (10)	0.2
IPSS, QoL improvement, mean (SD)	1.7 (1.2)	1.7 (1.3)	0.88
IPSS, total improvement, mean (SD)	8.3 (6.6)	10.2 (6.8)	0.31
Voiding subscore improvement, mean (SD)	7.1 (5.6)	7.4 (4.6)	0.72
Storage subscore improvement, mean (SD)	1.5 (3.1)	2.9 (3.5)	0.2
PVR decrease, ml, mean (SD)	30.6 (62.5)	26.4 (55.1)	0.88
Qₘₐₓ improvement, ml/s, mean (SD)	6.2 (7.6)	7.4 (8.1)	0.54
Enucleated tissue weight, g, mean (SD)	4.7 (3.7)	17 (13.7)	<0.001
Enucleation time, min, mean (SD)	19.8 (12.5)	39.3 (30.8)	0.04
Enucleated tissue ratio, %, mean (SD)	18.5 (13.9)	26.3 (14.4)	0.04

Abbreviations: see TABLE 1 and TABLE 2

TABLE 5 outlines HoLEP outcomes in both groups. There was no notable difference in Hb reduction between group 1 (mean [SD], 1.1 [1] g/dl) and group 2 (mean [SD], 0.7 [0.9] g/dl; *P* = 0.2). Both groups showed improvements in IPSS scores. Group 2 patients had slightly higer total IPSS score (mean [SD], 10.2 [6.8] vs 8.3 [6.6]) and storage subscores (mean [SD], 2.9 [3.5] vs 1.5 [3.1]), but these differences were insignificant. QoL improvements were similar in both groups (mean [SD], 1.7 [1.3] vs 1.7 [1.2]; *P *= 0.88).

Reduction in PVR was comparable (mean [SD], 30.6 [62.5] ml for prostate volume ≤40 ml vs 26.4 [55.1] ml for prostate volume >40 ml; *P *= 0.88). Improvement in Q_max_ was also similar (mean [SD], 6.2 [7.6] vs 7.4 [8.1] ml/s; *P *= 0.54). Group 2 patients required significantly longer enucleation times than those assigned to group 1 (mean [SD], 39.3 [30.8] vs 19.8 [12.5] min; *P *= 0.04), and they also had more tissue removed (mean [SD], 17 [13.7] vs 4.7 [3.7] g; *P *<⁠0.001). The enucleated tissue ratio was markedly higher in group 2 patients (mean [SD], 26.3% [14.4%] vs 18.5% [13.9%]; *P *= 0.04).

## DISCUSSION

HoLEP has emerged as a gold standard treatment for BPH, offering effective symptom relief across various prostate sizes. This study aimed to evaluate preoperative characteristics and postoperative outcomes in patients with prostate volume smaller than or equal to 40 ml and larger than 40 ml. The results showed that HoLEP significantly improved urinary symptoms, QoL, and functional parameters, regardless of the prostate size. The patients had better Q_max_ and PVR values, and their IPSS scores dropped considerably following the procedure. However, they were assessed over a 3-month follow-up period, which is relatively short and does not provide insights into the likelihood of future recurrence or complications.

Li et al^23^ explored a combination of thulium laser enucleation of the prostate (ThuLEP) and thulium fiber laser (TFL) for BPH cases complicated by bladder stones. They reported shorter surgery times, less pronounced Hb level decrease, and fewer catheterization days. QoL and IPSS scores improved, too. Compared with standard TURP with bladder lithotomy, this combination seemed to work better in those cases. They reported an impressive stone clearance rate of 100%.

Taking the above into account, we can conclude that HoLEP is highly effective, while ThuLEP combined with TFL is extraordinarily efficacious when bladder stones are involved. However, longer follow-up is needed, particularly in countries with fewer resources, such as Jordan, where these technologies could make a real difference.

A significant reduction in the IPSS scores and its subdomains underscore HoLEP’s efficacy in alleviating urinary obstruction and improving patient-reported outcomes. Prostate volume does not compromise the efficiency of the procedure in enhancing overall well-being, as seen in the similar QoL improvements in both groups.^1^**^,^**^16^

Although within clinically acceptable limits, the decrease in Hb levels was greater in the patients with larger prostates. This result is likely due to the increased enucleation time and a greater amount of tissue removed.^13^ Considerable improvements were observed in both PVR and Q_max_ scores. This demonstrates that HoLEP effectively addresses functional obstruction, regardless of the prostate size. However, larger prostates might present more significant initial obstructions, as shown in their slightly higher relative improvements.^12^**^,^**^14^

The results support the recommendation of HoLEP as the first-line treatment for large prostates, where other procedures may be less effective^1^**^,^**^13^

Although prostate size did not affect the effectiveness of HoLEP in the studied groups, the procedure involved removing more tissue and longer enucleation times for larger glands, which points to the need for a more focused preoperative planning.^1^**^,^**^8^

HoLEP results also proved to be more durable in comparison with more traditional options, such as TURP. In larger prostates especially, the outcomes tend to align with earlier findings,^11^**^,^**^12^ giving HoLEP a slight advantage in terms of consistent symptom relief. That is probably the reason why it is currently considered the preferred option in treating BPH^15^

Our study has certain limitations. Without extended follow-up, it is difficult to assess sustainability of the results and occurrence of potential complications. Also, the sample was quite small and all participants came from the same area, which precludes generalizability of the findings. Expanding the sample to other regions of Jordan or even beyond its borders would be advisable. Future work might also focus on other surgical approaches in order to draw long-term conclusions.

### Conclusions

The findings reaffirm the status of HoLEP as the gold standard for BPH treatment, as it offers durable symptom relief and excellent functional outcomes. Regardless of the prostatic volume, the patients exhibited significant improvements in urinary symptoms, QoL, and functional parameters.

## References

[BIBR-1] Elmansy H., Abbas L., Fathy M. (2024). Top‐down holmium laser enucle‐ ation of the prostate (HoLEP) versus traditional HoLEP for the treatment of benign prostatic hyperplasia (BPH): 1‐year outcomes of a randomized con‐ trolled trial. Prostate Cancer Prostatic Dis.

[BIBR-2] Bozzini G., Berti L., Aydoğan T.B. (2021). A prospective multicenter ran‐ domized comparison between holmium laser enucleation of the prostate (HoLEP) and thulium laser enucleation of the prostate (ThuLEP. World J Urol.

[BIBR-3] Daryanto B., Suryanullah W.S., Putra P.Y.P. (2025). Holmium laser enucleation of the prostate versus transurethral resection of the prostate in treatment of benign prostatic hyperplasia: a meta‐analysis of 13 randomized control tri‐ als. Curr Urol.

[BIBR-4] Zhao X., Jia L., Li W. (2025). Safety and efficacy of low‐powered holmium laser enucleation of the prostate in comparison with plasma kinetic resec‐ tion of prostate. Lasers Med Sci.

[BIBR-5] Xu J., Han B., Xia S., Jing Y. (2025). Beyond size: a comprehensive overview of small‐volume benign prostatic hyperplasia. Curr Urol.

[BIBR-6] Gao Z., Ding Y., Liu H. (2024). Comparative analysis of low‐power versus high‐power holmium laser enucleation of the prostate for symptomatic small‐volume benign prostatic hyperplasia: a prospective randomized con‐ trolled trial. J Int Med Res.

[BIBR-7] Chen Y.Y., Hua W.X., Huang Y.H. (2024). The safety and efficacy of five surgi‐ cal treatments in prostate enucleation: a network meta‐analysis. BMC Urol.

[BIBR-8] Budi A. (2024). A study analysis of the comparative effectiveness of transure‐ thral prostate resection and holmium laser enucleation in benign prostatic hyperplasia. Indones J Gen Med.

[BIBR-9] Kallidonis P., Spinos T., Peteinaris A. (2024). Salvage holmium laser enucle‐ ation of the prostate after previous interventions: a systematic review. BJU Int.

[BIBR-10] Bhatia A., Titus R., Porto J.G. (2024). Comparing outcomes of aquabla-tion versus holmium laser enucleation of the prostate in the treatment of be-nign prostatic hyperplasia: a network meta-analysis. BJUI Compass.

[BIBR-11] Choudhary M.K., Kolanukuduru K.P., Eraky A. (2025). Comparative outcomes of en‐bloc holmium laser enucleation of the prostate and transvesical robot‐assisted simple prostatectomy for the management of benign prostatic hy‐ perplasia: a propensity‐matched analysis. J Endourol.

[BIBR-12] Lee H., So S., Cho M.C. (2024). Clinical outcomes of holmium laser enucle-ation of the prostate: a large prospective registry-based patient cohort study under regular follow-up protocol. Investig Clin Urol.

[BIBR-13] Meng C., Peng L., Li J. (2022). Comparison of enucleation between thuli -um laser and holmium laser for benign prostatic hyperplasia: a systematic and meta-analysis. Asian J Surg.

[BIBR-14] Porreca A., Colicchia M., Tafuri A. (2022). Perioperative outcomes of hol -mium laser enucleation of the prostate: a systematic review. Urol Int.

[BIBR-15] Pirola G.M., Castellani D., Maggi M. (2022). Does power setting impact sur-gical outcomes of holmium laser enucleation of the prostate? A systematic review and meta-analysis. Cent Eur J Urol.

[BIBR-16] Liu K., Zong Y., Xiao R. (2025). Effect of laser energy consumption on early‐stage LUTS after HoLEP: comparison of symptom improvements between low and high energy consumed procedures. Curr Urol.

[BIBR-17] Oelke M., Bachmann A., Descazeaud A. (2013). EAU guidelines on the treatment and follow-up of non-neurogenic male lower urinary tract symptoms including benign prostatic obstruction. Eur Urol.

[BIBR-18] Barry M.J., Fowler F.J., O’Leary M.P. (2017). The American Urological Asso‐ ciation symptom index for benign prostatic hyperplasia. J Urol.

[BIBR-19] Abrams P.H., Griffiths D.J. (1979). The assessment of prostatic obstruc‐ tion from urodynamic measurements and from residual urine. Br J Urol.

[BIBR-20] Ballstaedt L., Leslie S.W., Woodbury B. Bladder post void residual vol‐ ume. In.

[BIBR-21] Fong K.Y., Gauhar V., Castellani D. (2024). Does concordance between pre‐ operatively measured prostate volume and enucleated weight predict out‐ comes in endoscopic enucleation of the prostate? Results from the REAP da‐ tabase. World J Urol.

[BIBR-22] Cornford P., Bergh R.C., Briers E. (2024). EAU‐EANM‐ESTRO‐ESUR‐ISUP‐SIOG guidelines on prostate cancer—2024 update. Part I: screen‐ ing, diagnosis, and local treatment with curative intent. Eur Urol.

[BIBR-23] Li Y., Yang Y., Chen J. (2024). Thulium laser enucleation of the prostate plus thulium fiber laser therapy for benign prostatic hyperplasia combined with bladder stones. Wideochir Inne Tech Maloinwazyjne.

